# Rootstock identity in melon-pumpkin graft combinations determines fruit metabolite profile

**DOI:** 10.3389/fpls.2022.1024588

**Published:** 2023-01-25

**Authors:** Maria Dolores Camalle, Shimon Pivonia, Udi Zurgil, Aaron Fait, Noemi Tel-Zur

**Affiliations:** ^1^ The Albert Katz International School for Desert Studies, The Jacob Blaustein Institutes for Desert Research, Ben-Gurion University of the Negev, Sede Boqer, Israel; ^2^ Arava Research and Development Center, Yair Experimental Station, Central and Northern Arava, Hazeva, Israel; ^3^ French Associates Institute for Agriculture and Biotechnology of Drylands, Blaustein Institutes for Desert Research, Ben-Gurion University of the Negev, Sede Boqer, Israel

**Keywords:** amino acids, flavonoids, fruit metabolites, graft incompatibility, sugars, yield

## Abstract

Grafting has the potential to improve melon fruit yield and quality, but it is currently held that a lack of compatibility between the rootstock and scion compromises such an effect. To throw light on this subject, we studied melon-pumpkin graft combinations with different levels of compatibility to assess to the effect of the rootstock identity on melon fruit yield and quality, including total fruit *ortho*-diphenols, total flavonoids, and primary fruit metabolites. Melon cv. ‘Kiran’ (Ki) was grafted onto three pumpkin rootstocks, ‘TZ-148’ (TZ), ‘Shimshon’ (Sh), and ‘53006’ (r53), characterized by high, moderate, and low compatibility, respectively. The non-grafted melon cultivar Ki was used as the control. The incompatible combination Ki/r53 gave the lowest fruit yield and the lowest average fruit weight. In that combination, the content of total *ortho*-diphenols increased vs. Ki and Ki/TZ and that of total flavonoids decreased vs. Ki/Sh. In addition, concentrations of the amino acids, glutamate, methionine, valine, alanine, glycine, and serine, increased in the pulp of the two compatible combinations, i.e., Ki/TZ and Ki/Sh, suggesting that rootstock identity and compatibility with melon Ki scion modulated amino acid synthesis. Our results show an association between rootstock identity (and level of compatibility with the scion) and an enhancement of fruit nutritional values, i.e., high concentrations of organic acids (determined as citrate, malate, fumarate, and succinate) and soluble carbohydrates (sucrose) were recorded in the pulp of the two compatible combinations, i.e., Ki/TZ and Ki/Sh.

## Introduction

Grafting – a horticultural technique used for plant propagation – constitutes an essential part of modern agriculture and hence makes an important contribution to food security worldwide. This technique combines traits from two selected plant parts, i.e., the shoot (also known as the scion) and the root (also, rootstock), to generate superior combinations. Rootstock traits, for example, can confer better tolerance to biotic and abiotic stress (Edelstein et al., 2005; Martinez-Andujar et al., 2016; Edelstein et al., 2017), and they can also enable growers to overcome certain cultivation challenges, such as improving yields and enhancing nutrient contents of fruits and vegetables (Colla et al., 2015; Kyriacou et al., 2017). However, for certain crops, there are no suitable rootstocks, and agriculturalists therefore use rootstock from a different species or genus.

In melon (*Cucumis melon* L.), grafting is used to facilitate production under challenging biotic and abiotic conditions (Cohen et al., 2002; Colla et al., 2006; Davis et al., 2008; Louws et al., 2010; Rouphael et al., 2016). For example, the low tolerance of some melon cultivars to environmental stressors limits plant growth and adversely affects crop yield and quality (Edelstein et al., 2004; Cohen et al., 2007; Edelstein et al., 2011; Edelstein et al., 2017). Grafting such sensitive melon cultivars onto tolerant interspecific pumpkin rootstock, i,e., *Cucurbita maxima* × *Cucurbita moschata*, allows cultivation and production under a broad spectrum of adverse environmental conditions (Nisini et al., 2002; Cohen et al., 2007; Fita et al., 2015). However, the resulting melon-pumpkin grafts are not always successful, possibly due to rootstock–scion incompatibility, and sometimes the grafting may even compromise fruit yield and quality (Edelstein et al., 2004; Cohen et al., 2007; Minuto et al., 2009; Soteriou et al., 2016).

Melon quality, like that of all fruit, involves a complex interplay of flavor, texture, and appearance. In melon, four groups of metabolites contribute to fruit quality. The first group comprises various sugars, with sucrose being the dominant sugar providing sweetness in the ripe melon (Burger et al., 2000; Vallone et al., 2013). The second group comprises amino acids, compounds that are essential for human health. These compounds may also significantly affect fruit flavor, since they are the precursors of secondary metabolites, including aroma compounds (Wang et al., 1996; Gonda et al., 2010; Gonda et al., 2013). The third group comprises polyphenols, which are antioxidants, conferring protection on the fruit from pathogenic attacks (Sudheeran et al., 2020) and on humans from reactive oxygen species (Sroka and Cisowski, 2003). The final group of compounds are the organic acids, including citrate and malate, which – at moderate concentrations – contribute to the taste of the fruit (Burger et al., 2003). However, there appears to be a lack of consensus on the effect of grafting on the quality and yields of fruit from various melon-pumpkin grafts. For example, one study reported an increase in amino acids, including threonine, glutamate, methionine, isoleucine and lysine, in the fruit of melon-pumpkin grafted plants (Liu et al., 2010), and another showed that total free amino acids and soluble proteins were higher in fruits of non-grafted melons (Ruiz and Romero, 1999). Yet another study found a significant increase in two flavonoids, i.e., pelargonidin 3-(6’ ‘-malonylglucoside)-5-glucoside and malvidin 3-rutinoside, in the fruits of grafted plants (Zhang et al., 2019), a finding that indicated that rootstock–scion compatibility facilitates the accumulation of fruit metabolites. In parallel, there are discrepancies in reports of fruit yield; for example, Condurso et al. (2012) reported a higher yield from grafted than from non-grafted plants, while other studies reported no differences in fruit yield between different melon–pumpkin combinations and non-grafted plants (Traka-Mavrona et al., 2000).

A careful reading of the studies on melon-pumpkin grafts indicates that differences in fruit quality and yield could be associated both with the particular rootstock–scion combination and also with environmental factors and growing season (Soteriou et al., 2016). Therefore, our working hypothesis in the current study was that fruit metabolites, including amino acids, sugars, organic acids, and polyphenols in the melon pulp, are dictated by rootstock identity and by the level of rootstock–scion compatibility. We thus sought to determine whether rootstock–scion compatibility modulates fruit quality by comparing fruit metabolite profiles between the plants of three different graft combinations (with different levels of compatibility) vs. non-grafted plants. In addition, differences in the yields between the three different graft combinations were evaluated. Based on a GC-MS analysis of melon pulp metabolites, we did indeed identify different concentrations of fruit metabolites in the three graft combinations and the non-grafted plants, confirming that rootstock–scion compatibility modulated the accumulation of fruit metabolites.

## Materials and methods

### Plant material

Melon (*Cucumis melon* L.) cv. ‘Kiran’ (Ki), selected for its fruit quality (particularly sweetness), was grafted onto three pumpkin (*Cucurbita maxima* Duch. × *Cucurbita moschata* Duch.) rootstocks (two commercial and one experimental) with different degrees of compatibility with the scion (S. Pivonia, unpublished data), namely, the highly compatible TZ-148’ (TZ), the moderately compatible ‘Shimshon’ (Sh), and ‘53006’ (r53), an experimental rootstock that was shown to have low compatibility with Ki, since the plants collapsed at fruit load (S. Pivonia, personal communication). The plants were grafted by Hishtil Nurseries Co. (Ashkelon, Israel) using the whip grafting procedure described in Camalle et al. (2021).

### Growing conditions

The experiment was conducted in the Arava valley, Israel, from December 2016 to March 2017. Forty days after grafting, the plants were transferred to a nethouse. Plants (n=5) of each combination, i.e., Ki/r53, Ki/Sh, and Ki/TZ, and also non-grafted Ki plants (as control) were randomly distributed in four blocks. The average temperature in the nethouse was 39/25°C, and the length of daylight changed from about 10 h in December to about 12 h in March. Plants were fertigated twice a daily, with a total volume of 1.5 L containing 0.1% of N, P, K until fruit set; thereafter, the irrigation water was supplemented with 0.15% N, P, K + microelements (Fe, Mn, Zn, Cu and Mo) (7-3-7 Gat NPK*).

### Fruit harvest, yield calculation, and sample preparation

The fruit (5 fruits per block per graft combination + non-grafted, giving a total of 20 fruits per combination) was harvested at the mature commercial stage in March 2017 and transported on ice to the laboratory. The fruits were weighed, and average fruit weight was calculated.

The total yield was quantified as total fruit/m^2^ and was expressed as kg/m^2^. Additionally, fruit yield from non-grafted Ki, Ki/r53, and Ki/TZ in 2017-2018 was added.

For metabolomics profiling, fruits of the three graft combinations and non-grafted plants were peeled, and the pulp was cut into small pieces of approximate size 2 cm × 2 cm. Pulp pieces from five fruits per block for each graft combination and for non-grafted plants were pooled and considered as a single biological replicate (four replicates, one per block, were used per combination). Approximately 25 g of each pooled sample were flash-frozen in liquid nitrogen and stored at -80°C prior to lyophilization. The four biological replicates of lyophilized samples were used for quantification of total *ortho*-diphenols and total flavonoids and for metabolomics profiling.

### Extraction and quantification of *ortho*-diphenols and total flavonoids

For each rootstock–scion combination and for the non-grafted plants, 400 mg of lyophilized pulp powder were extracted in 1 ml of methanol, vortexed for 10 s, heated for 10 min at 60°C, and vortexed for 10 s. Samples were then centrifuged at 14,000 rpm for 5 min at room temperature, and *ortho*-diphenols and flavonoids were determined in the supernatant. For quantification of *ortho*-diphenols, 100 μl of the supernatant were mixed with 380 μl of Milli-Q water and 320 μl of 100 mM phosphate buffer (pH 6.5). Thereafter, 640 μl of Na_2_MoO_4_ and the samples were held at room temperature for 15 min. The absorbance was measured against a blank of 100 μl of methanol at 370 nm. Readings were calibrated against a standard curve of known concentrations of caffeic acid, and the content of *ortho*-diphenols was expressed as caffeic acid equivalents per gram of pulp.

The quantification of total flavonoids was performed as described by Simirgiotis et al. (2008) with minor modifications: 200 μl of the supernatant was mixed with 300 μl of Milli-Q water and 40 µl of 5% NaNO_2_ and left to stand for 5 min. Thereafter, 40 µl of 10% AlCl_3_ in methanol were added, and the mixture was left to stand for another 5 min. Then, 200 μl of 1 M NaOH solution and 50 μl of Milli-Q water were added. The absorbance was read immediately at 510 nm against a blank comprising 200 μl of methanol. Readings were calibrated against a standard curve of known concentrations of quercetin. The concentration of total flavonoids was expressed as quercetin equivalents per gram of sample.

### Metabolite extraction and derivatization for GC-MS

To study the effect of rootstock–scion compatibility on fruit pulp metabolites, the metabolite profile, including amino acids, sugars, and organic acids, was determined in 50 mg lyophilized pulp powder, as described by Lisec et al. (2006). Briefly, the powder was dissolved in 1 ml of cold extraction solution [methanol: chloroform: water (2.5:1:1)]; 200 µl of 1 mg/ml [^13^C_6_] sorbitol (used as the internal standard) was added; and the solution was vortexed for 10 s. The mixture was then incubated in an orbital shaker for 10 min at 25°C and sonicated for 10 min at room temperature. The homogenate was centrifuged at 14,000 rpm at 4°C for 10 min. The supernatant was transferred to a new tube with 300 µl of chloroform and 300 µl of MiliQ water, vortexed for 10 s, and centrifuged again at 14,000 rpm for 10 min at 4°C. Finally, 50 µl of the aqueous phase was dried under vacuum and then derivatized for gas chromatography-mass spectrometry (GC-MS) analysis, as described in Lisec et al. (2006). The derivatized samples (0.5 µl) were injected into a GC–MS system consisting of a 7693 autosampler, a 7890B GC, and a 5977B single quadrupole mass spectrometer (Agilent Ltd). GC-MS conditions were as described previously (Reshef et al., 2019). For quantifying the concentrations of sugars, organic acids, and amino acids, 1 ng of a mixed stock standard consisting of the following compounds in methanol and MiliQ water was prepared and analyzed by gas chromatography: alanine, asparagine, glutamate, glutamine, glycine, methionine, proline, serine, valine, phenylalanine, sucrose, raffinose, trehalose, fructose, malate, fumarate, succinate, and citrate. The mixed stock standard was diluted to eight different levels. The standard mixture (1 µl) was injected into a GC–MS system consisting of a 7693 autosampler, a 7890B GC, and a 5977B single quadrupole mass spectrometer (Agilent Ltd). All standards were acquired from Sigma-Aldrich (Sigma-Aldrich Israel, Rehovot, Israel; https://www.sigmaaldrich.com/israel.html).

### Statistical analysis

To test the differences between graft combinations, the data set was subjected to a one-way ANOVA analysis. Where ANOVA indicated significant differences at *p*< 0.05, means were compared by the Tukey-Kramer test. A data set of grafted and non-grafted fruit was subjected to principal component analysis (PCA) to determine variability among the graft combinations and non-grafted melon plants. The relative response ratios were normalized to the median of each metabolite across all samples. The resulting value was then transformed into its log_2_ value. The PCA was performed according to Kassambara and Mundt (2017), and a heat map was obtained with the “ComplexHeatmap” package (Gu et al., 2016) where the metabolites were grouped into different clusters according to their relative abundance. All statistical analyses were performed using R Statistical Software (version 3.6.3). Normalized data was uploaded into MetaboAnalyst 5.0 for modeling using partial least squares discriminant analysis (PLS-DA). Variable importance of projection (VIP) scores were calculated for each metabolite in the graft combinations and non-grafted plants for the degree of discrimination within the PLS-DA model.

## Results

### Effect rootstock–scion compatibility on fruit yield and fruit weight

**Figure 1:**
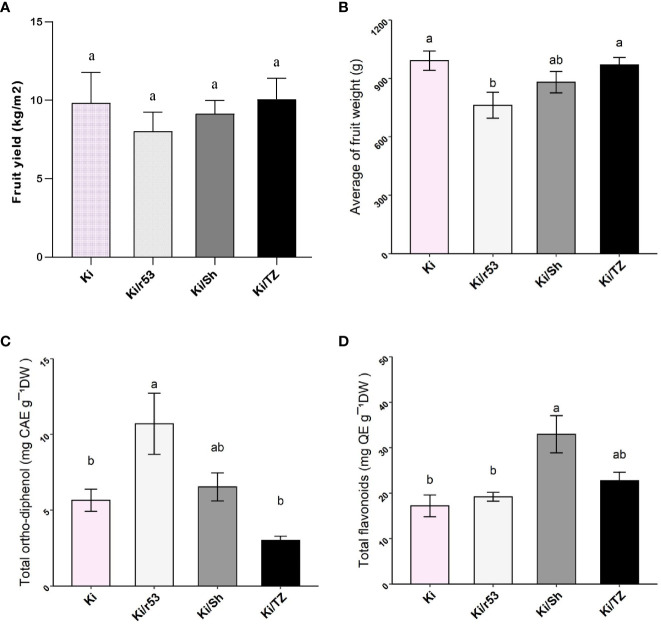
Effect of rootstock on fruit yield and weight, total *ortho*-diphenols and total flavonoids of grafted and non-grafted melon plants **(A)** Fruit yield from non-grafted Ki and grafted Ki/53, Ki/Sh and Ki/TZ (kg/m²). **(B)** Fruit weight from grafted and non-grafted melon plants (g). **(C)** Content of total *ortho*-diphenols expressed as caffeic acid equivalents per gram of pulp (CAE). **(D)** Content of total flavonoids expressed as quercetin equivalents per gram of pulp (QE). Statistical comparisons between graft combinations and non-grafted plants were performed by one-way ANOVA, followed by Tukey’s *post hoc* test. Different letters indicate significant differences (*p < 0.05*) between grafted and non-grafted plants.

Fruit yield was calculated for the non-grafted Ki and three rootstock–scion combinations as kilograms per square meter. Unfortunately, several plants of the incompatible combination, i.e., Ki/r53, collapsed before harvest, resulting in a decrease in the number of plants; therefore, only areas with the same number of plants per square meter were included in the calculation. Notably, the incompatible combination Ki/r53 gave the lowest fruit yield, but the yield was statistically similar to the others (**Figure 1A**). Yield (average ± SE) calculated for the 2017-2018 harvesting season was 9,83 ± 1.12 kg/m^2^ for the non-grafted Ki, 8,03 ± 0,77 kg/m^2^ for Ki/r53, and 10,07 ± 0,77 kg/m^2^ Ki/TZ. No significant statistical differences were observed between the non-grafted and grafted plants in terms of yield.

Interestingly, no statistically significant differences were observed in average fruit weight between the two compatible combinations, i.e., Ki/TZ and Ki/Sh, and the non-grafted plants, but fruit weight for the incompatible Ki/r53 was significantly lower (**Figure 1B**). These results show that rootstock–scion compatibility does indeed affect fruit weight and yield.

### Effect of rootstock–scion compatibility on contents of total *ortho*-diphenols and total flavonoids in the fruit pulp

The content of total *ortho*-diphenols, expressed as caffeic acid equivalents per gram of pulp, was significantly higher in the pulp of Ki/r53 (**Figure 1C**). No statistically significant differences were observed between Ki/TZ, Ki/Sh and non-grafted plants (**Figure 1C**). The content of total flavonoids, expressed as quercetin equivalents per gram, was significantly higher in the pulp of Ki/Sh, and concentrations were statistically similar in non-grafted, Ki/TZ and Ki/r53 fruit (**Figure 1D**).

### Rootstock genotype modulates pulp metabolites

**Figure 2:**
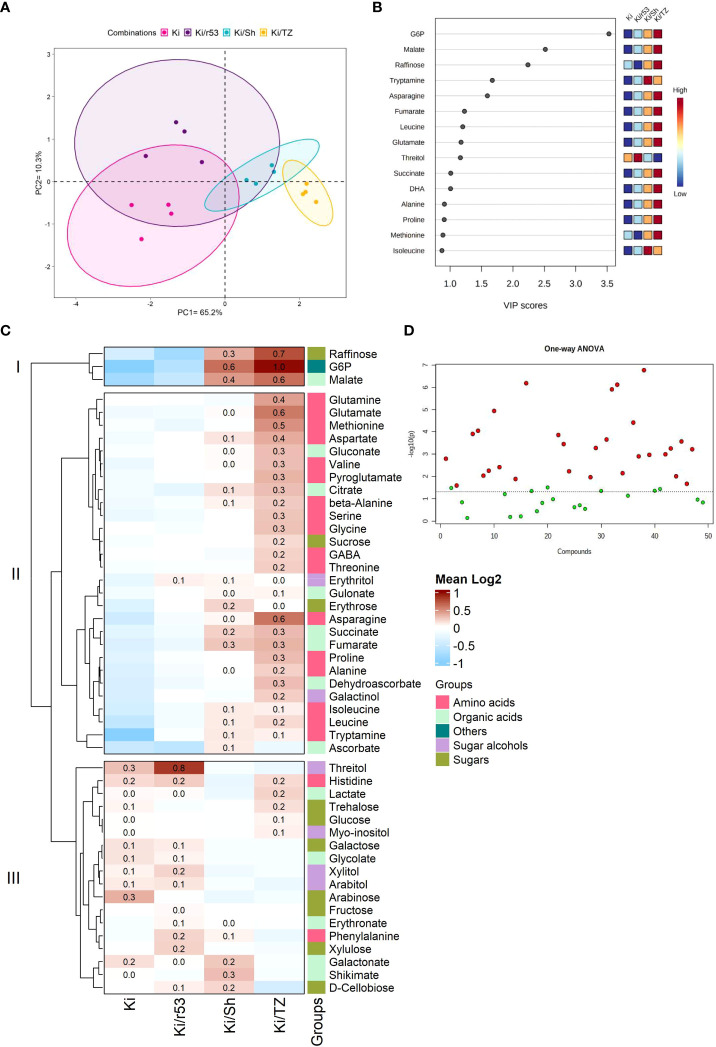
Pulp metabolite profiling of the fruit of grafted (Ki/r53, Ki/Sh and Ki/TZ) and non-grafted (Ki) melon plants (n=4). **(A)** PCA plot of GC-MS pulp metabolomic profile; each dot represents one biological replicate. **(B)** Partial least squares discriminant analysis (PLS-DA) variable importance in projection (VIP) of the first component, indicating the most abundant metabolites in descending order of importance, VIP scores were determined using MetaboAnalyst 5.0. **(C)** Heat map showing the hierarchical clustering of the metabolite log2 fold-change values by Ward’s method. Dark red indicates a high relative abundance of the metabolites, and light blue indicates a low fold change. **(D)** Statistical comparisons between graft combinations and non-grafted plants were performed by one-way ANOVA, followed by Tukey’s *post-hoc* test. Red dots represent metabolites that were significantly different between the fruit of grafted and non-grafted plants (p < 0.05), and green dots represent metabolites that were not significantly different between the fruit of grafted and non-grafted plants.

A comprehensive analysis of fruit pulp metabolites was performed to elucidate the effect of the rootstock–scion combination on fruit biochemical parameters. The pulp of non-grafted Ki was used as the control to evaluate the relationship between the degree of graft compatibility and fruit pulp quality. Metabolite profile-based PCA was used to group graft combinations, explaining 65.2% and 10.3% of data variance (**Figure 2A**). VIP scores (which estimate the importance of each variable) showed that the relative abundances of three metabolites, i.e., glucose-6-phosphate (GP6), followed by malate and raffinose, were present in higher concentrations in the fruit of the compatible combination, i.e., Ki/TZ (**Figure 2B**), thus suggesting that these compounds are the variables of the highest importance in this model. Tryptamine abundance was found to have the same impact in the model as asparagine, i.e., VIP scores for these two metabolites were the same. Similarly, the abundances of threitol, fumarate, leucine, and glutamate had the same impact, as did the abundances of isoleucine, alanine, proline, and methionine (**Figure 2B**).

Fruit metabolite profiling revealed an enhancement of the relative abundances of certain pulp metabolites (amino acids, sugars, and organic acids). These included high relative abundances of raffinose, G6P and malate in the two compatible combinations Ki/Sh and Ki/TZ vs. the fruit pulp of Ki/r53 and of the non-grafted plants [Cluster I (**Figure 2C**)]. High relative abundances of several amino acids, including glutamate, glutamine, methionine, aspartate, valine, serine, glycine, GABA, and proline, were observed in the pulp of the compatible Ki/TZ combination [Cluster II (**Figure 2C**)]. The mean relative abundances of threitol, phenylalanine, xylulose and d-cellobiose were higher in the Ki/r53 pulp than in the non-grafted pulp [Cluster III (**Figure 2C**)]. On the other hand, the mean relative abundances of galactose, glycolate, arabitol, and lactate were similar in the pulp of Ki/r53 and of the non-grafted plants [Cluster III (**Figure 2C**)]. The above-described results showed different relative abundances of several metabolites in the pulp of fruit from the different graft combinations and non-grafted Ki. Further studies are needed to assess to the relationship between graft compatibility and its effect of fruit metabolism, which may have a marked effect on fruit quality and flavor.

Overall, one-way ANOVA showed that 29 of the 49 metabolites detected were significantly different in the fruits of grafted and non-grafted plants (**Figure 2D**). These results provide empirical evidence that rootstock identity had a strong effect in the accumulation of 29 metabolites but no effect in the other 20 metabolites, suggesting that the level of rootstock–scion compatibility significantly influences the regulation of fruit metabolites.

### The level of rootstock–scion compatibility affects the concentrations of sugars, organic acids, and amino acids in the pulp

Since the pulp of Ki/TZ showed relatively higher contents of sugars, organic acids, and amino acids, the absolute values of these compounds were calibrated against standard curves with known concentrations of the particular metabolite (**Table 1**). One-way ANOVA analysis showed that sucrose, raffinose, and trehalose concentrations were significantly higher in the pulp of Ki/TZ than in Ki/Sh, Ki/r53, and non-grafted pulp. In addition, the fructose concentration was significantly higher in the pulp of Ki/r53.

**Table 1:** Concentrations of pulp sugars, organic acids, and amino acids in melons of grafted and non-grafted plants (n = 4).

Metabolite	Content (mg g⁻¹DW)			
	Ki	Ki/r53	Ki/Sh	Ki/TZ
Sugars				
Sucrose	128.09 ± 3.52 ^b^	130.96 ± 2.43 ^b^	132.104 ± 3.96 ^ab^	145.29 ± 3.49 ^a^
Fructose	45.84 ± 0.83 ^b^	52.91 ± 2.20 ^a^	44.96 ± 2.05 ^b^	44.75 ± 1.09 ^b^
Trehalose	19.67 ± 1.13 ^b^	17.96 ± 0.71 ^b^	20.32 ± 0.57 ^b^	24.51 ± .92 ^a^
Raffinose	1.61 ± 0.11 ^b^	1.63 ± 0.16 ^b^	1.85 ± 0.13 ^b^	2.61 ± .11 ^a^
Organic acids				
Citrate	24.45 ± 1.96 ^b^	24.77 ± 1.06 ^b^	32.71 ± .77 ^a^	39.50 ± 2.70 ^a^
Fumarate	8.87 ± .25 ^b^	9.31 ± .82 ^b^	13.91 ± .40 ^a^	15.13 ± .77 ^a^
Malate	3.19 ± 0.19 ^b^	4.22 ± 0.68 ^b^	9.26 ± 1.07 ^a^	10.97 ± 0.63 ^a^
Succinate	0.94 ± 0.19 ^b^	1.08 ± 0.23 ^b^	1.52 ± 0.14 ^a^	2.24 ± 0.26 ^a^
Amino acids				
Glutamate	1.63 ± 0.13 ^b^	1.93 ± 0.13 ^b^	2.39 ± 0.20 ^ab^	3.08 ± 0.23 ^a^
Methionine	2.14 ± 0.34 ^b^	1.58 ± 0.14 ^b^	2.42 ± 0.16 ^ab^	3.09 ± 0.07 ^a^
Valine	1.64 ± 0.10 ^b^	2.05 ± 0.04 ^b^	2.47 ± 0.25 ^ab^	2.87 ± 0.24 ^a^
Proline	2.07 ± 0.04 ^c^	2.38 ± 0.09 ^b^	2.39 ± 0.09 ^b^	2.69 ± 0.03 ^a^
Phenylalanine	2.11 ± 0.02 ^b^	2.60 ± 0.11 ^a^	2.22 ± 0.09 ^ab^	2.36 ± 0.12 ^ab^
Alanine	1.65 ± 0.13 ^b^	1.75 ± 0.11 ^b^	2.04 ± 0.05 ^ab^	2.37 ± 0.10 ^a^
Glycine	1.49 ± 0.11 ^ab^	1.41 ± 0.11 ^b^	1.46 ± 0.08 ^ab^	1.80 ± 0.02 ^a^
Glutamine	1.117 ± 0.15 ^b^	1.11 ± 0.07 ^b^	1.15 ± 0.05 ^b^	1.708 ± 0.05 ^a^
Asparagine	0.57 ± 0.02 ^b^	0.64 ± 0.02 ^b^	0.60 ± 0.02 ^b^	0.78 ± 0.03 ^a^
Serine	0.16 ± 30.01 ^b^	0.15 ± 0.01 ^b^	0.18 ± 0.01 ^ab^	0.21 ± 0.005 ^a^

Different letters indicate significant differences (p < 0.05) between grafted and non-grafted plants (one-way ANOVA, followed by Tukey’s post-hoc test).

For the organic acids, there were significant differences in pulp concentrations between the different graft combinations and also vs. the non-grafted plants (**Table 1**). For example, citrate, fumarate, malate, and succinate contents were significantly higher in the two compatible combinations, Ki/TZ and Ki/Sh, than in the incompatible combination Ki/r53 and in the non-grafted plants.

For the amino acids, there was a significantly higher accumulation of glutamate, methionine, valine, proline, alanine, glutamine, asparagine, and serine in the pulp of Ki/TZ than in the pulp of Ki/r53 and the non-grafted plants (**Table 1**). In contrast, the pulp of Ki/Sh and Ki/TZ exhibited similar concentrations of these amino acids, with the exceptions of proline, glutamine, and asparagine, which were significantly higher in the pulp of Ki/TZ (**Table 1**). Notably, the pulp of Ki/r53 showed a higher phenylalanine concentration than the pulp of the Ki plants but similar concentrations to Ki/TZ and Ki/Sh (**Table 1**). These results provided evidence for the effect of the rootstock–scion combination on the contents of amino acids, sugars, and organic acids in the fruit pulp.

## Discussion

### The level of rootstock–scion compatibility modulates fruit yield and concentrations of polyphenols

The use of grafted plants has dramatically increased in horticulture due to the growing demand for higher yields and for increased tolerance to biotic and abiotic stress (Davis et al., 2008; Schwarz et al., 2010; Condurso et al., 2012). Nevertheless, for melon scion grafted onto different Cucurbita rootstocks there does not seem to be a clear picture of which graft combinations give the optimal results in terms of yield and fruit quality (Edelstein et al., 2005; Colla et al., 2015). Using three rootstocks with different degrees of compatibility to the commercial melon scion, our study showed that rootstock–scion incompatibility elicited a decrease in fruit yield, i.e., the lowest yield was obtained for the incompatible graft combination, Ki/r53 (**Figure 1A**). Lower yield was also observed for Ki/53 during the subsequent harvesting season, even though the results were not statistically significant. Similar average fruit weights were recorded for the two compatible combinations, i.e., Ki/TZ and Ki/Sh, and the non-grafted plants (**Figure 1B**). Traka-Mavrona et al. (2000) and Condurso et al. (2012) reported no differences in the fruit yield between grafted and non-grafted melon plants. Thus, it appears that compatible rootstocks can indeed produce similar yields and similar fruit weights to those of non-grafted melons, but a compatible rootstock enhanced fruit quality, as was shown by the enhancement of some fruit metabolites. The results of this study, in which fruit yield was compared between graft combinations, suggest an association between an incompatible rootstock and reduced yield.

It has been shown that *ortho*-diphenolic compounds, i.e., catechin, epicatechin, quercetin, and gallic, caffeic and tannic acids, are abundant during the green stage of different fruit but decrease sharply during fruit maturation (Rustioni and Failla, 2016), while flavonoids accumulate in high concentrations during the ripening stage (Mokhtar et al., 2021). We therefore determined the contents of total *ortho*-diphenolics and total flavonoids as indicators of whether the level of rootstock–scion compatibility modulates the concentrations of these compounds. Our results revealed that the contents of total *ortho*-diphenols and total flavonoids in the pulp varied between the different graft combinations (**Figures 1C, D**). For example, *ortho*-diphenols were the most abundant phenolic compounds in the incompatible combination Ki/r53, but they were present in low abundance in the pulp of both non-grafted melons and melons of the Ki/TZ compatible combination (**Figure 1C**). Rustioni and Failla (2016) published a study in grapes showing that the decrease of *ortho*-diphenol levels was associated with fruit ripening. Our findings showing the abundance of *ortho*-diphenols in Ki/r53 may thus suggest that under the incompatible rootstock the natural process of fruit maturation is altered but they could also indicate that other stressors affect the fruit parameters and performances in the incompatible combination. To the best of our knowledge, the current study is the first to report the concentrations of *ortho*-diphenols in melon pulp and further studies are needed to assess to the effect of the level of scion-rootstock compatibility in the process of fruit maturation.

We also determined the content of total flavonoids present in the fruits, since – being antioxidant compounds – they have important health value for humans (Kumar and Pandey, 2013). The fruit pulp of Ki/Sh exhibited the highest concentration of total flavonoids, followed by Ki/TZ, but the difference between the two was not statistically significant (**Figure 1D**). The relationship between total flavonoids in melon fruit and the level of scion-rootstock compatibility of the graft combinations used in this study was beyond the scope of our study, but it is worthy of mention that flavonoid biosynthesis is often enhanced under stress, e.g., high concentrations of quercetin are considered to confer photoprotection (Ryan et al., 2002; Treutter, 2005). Moreover, flavonoids play a significant role in the postharvest shelf life of fruits and vegetables (Lattanzio, 2003; Sivankalyani et al., 2016). Further research is needed to assess to the role of the higher accumulation of flavonoids in the fruit pulp of Ki/Sh and Ki/TZ observed in this study, which might be associated with a better tolerance of those graft combinations to environmental stressors and/or the maintenance of fruit quality upon storage.

### The level of rootstock–scion compatibility affects the concentrations of pulp metabolites

Our data point to a possible relationship between the level of rootstock–scion compatibility and the relative abundance of certain metabolites in the pulp (**Figure 2**). For example, multivariant PLS-DA analysis (in which the closer the VIP score to ≥ 1, the more important the variable) demonstrated that GP6 had high importance in the model for fruit pulp of Ki/TZ. Considering that G6P can be used for sucrose synthesis (Ruan, 2014), a high G6P content in the pulp can perhaps serve as a biomarker for the level of rootstock–scion compatibility.

As mentioned above, fruit quality is a complex manifestation of a combination of several parameters, with the concentrations of sugars, organic acids, and amino acids being among the key metabolites determining fruit organoleptic characteristics (Kader, 2008; Egydio et al., 2013). Our results did indeed show important differences in the abundance of specific metabolites in the pulp between the studied rootstock–scion combinations, which, in turn, might affect fruit quality. For most fruits, flavor is determined by the balance between acidity and sweetness, complemented by volatile compounds (Burger et al., 2009; Cohen et al., 2012), with fructose, glucose and sucrose being the major sugars contributing to sweetness (Linge and Dunler, 1987; Burger et al., 2000). In melon, sweetness is determined largely by the accumulation of sucrose during the final fruit ripening stage (Burger et al., 2000; Burger and Schaffer, 2007), with a low sugar content giving poor fruit quality (Yamaguchi et al., 1977). A review of previous studies together with the results of the current study – as elaborated below – suggests that grafting melon scion onto a compatible rootstock might contribute to overcoming the quality problem of a lack of sweetness. A previous study showed, for example, a low sucrose content in the fruit of the ‘Raymond’ scion grafted onto TZ rootstock (Soteriou et al., 2016), whereas our results showed high sucrose accumulation in the fruit pulp of Ki grafted onto TZ. Surprisingly, the content of one of the pulp sugars, i.e., fructose, was higher for the Ki scion grafted onto r53 rootstock than for the other plants, suggesting that the lower level of sucrose was probably due to the rapid hydrolysis of sucrose into fructose. Organoleptic evaluation was beyond of the scope of this study. However, preliminary tests indicated that Ki/TZ fruits were the most tasty and had a strong aroma, probably because of the higher sucrose levels in this graft combination. In contrast, fruits from the non-grafted Ki plants appeared to be slightly less sweet than those of the grafted plants.

Organic acids in the pulp influence fruit, senescence, storage performance and also flavor, with moderate concentrations of organic acids being likely to enhance the unique flavor of melon (Burger et al., 2003). In melon fruit, citrate is the major organic acid, accounting for about 0.2% of the fresh weight (Wang et al., 1996). Notably, in our study, citrate and malate levels were significantly higher in the fruit pulp of the compatible combinations, Ki/TZ and Ki/Sh (**Table 1**), with the higher levels probably enhancing both fruit flavor and storage performance. Our findings for organic acids thus provide yet another indication of the beneficial effect of grafting melon scion onto a compatible rootstock.

In our study, the amino acids, asparagine, aspartate, glutamate, glutamine, glycine, methionine, proline, and valine, were highest in the fruit pulp of the compatible Ki/TZ combination (**Table 1**). These results are consistent with those of the study of Liu et al. (2010) on different melon–pumpkin graft combinations. Of particular interest are methionine, valine and glutamate. The former two amino acids are metabolized into aroma compounds (Gonda et al., 2010; Gonda et al., 2013), and thus increased concentrations of these of aroma-compound precursors in Ki/TZ fruits might indicate that the unique aroma of melon fruits can be improved by grafting the scion onto a compatible rootstock. We have shown that the contents of amino acids in the pulp may be modulated by the graft combination. Therefore, it is vital to identify the rootstock–scion combination giving optimal enhancement of specific amino acids in the pulp.

## Conclusions

Overall, our results showed that in compatible rootstock–scion combinations concentrations of fruit metabolites were enhanced, whereas in incompatible combinations, fruit metabolites were similar to those of non-grafted plants. In particular, we showed that the identity of the rootstock might modulate fruit metabolites, e.g., compatible rootstocks enhanced the concentrations of glutamine, methionine, valine, alanine, glycine, serine, sucrose, citrate, malate, and fumarate. In the incompatible combination, pulp metabolites remained similar to those in non-grafted plants, suggesting that grafting onto an appropriate rootstock is a prerequisite for improving the abundance of specific metabolites. Alongside the enhancements in metabolite concentrations in the compatible combinations, reduced average fruit weight was observed in the incompatible rootstock r53. We stress the need to detect graft incompatibility in the early stages of the selection, since only compatible rootstock–scion combinations will facilitate an enhancement of fruit quality, especially in terms of concentrations of amino acids, sugars, and organic acids, along with increasing yield and fruit weight, thereby offering higher profits for farmers and improved nutritional value for consumers.

## Data availability statement

The raw data supporting the conclusions of this article will be made available by the authors, without undue reservation.

## Author contributions

MC, AF, and NT-Z conceived and planned the study. MC led and conducted the research. SP designed and conducted a nethouse experiment. UZ contributed to the trial and data analysis. MC, AF and NT-Z were responsible for data curation. AF supervised the metabolomics analysis. MC wrote the original draft with AF and NT-Z. All authors contributed to the article and approved the submitted version.
